# Intraoperative Stenting of Hepatic Ducts to Aid Dissection in an 8-Month-Old Child with Large Hepatoblastoma and Severe Porta Distortion

**DOI:** 10.1007/s13193-025-02386-6

**Published:** 2025-08-04

**Authors:** Sajid S. Qureshi, Abdeali Saif Arif Kaderi, Samreen S. Qureshi

**Affiliations:** 1 Division of Paediatric Surgical Oncology, Department of Surgical Oncology, Tata Memorial Hospital and Advanced Centre for Training Research and Education in Cancer (ACTREC), Tata Memorial Centre, Ernest Borges Road, Parel, Mumbai, 400012 India; 2 Homi Bhabha National Institute (HBNI), Mumbai, India

**Keywords:** Hepatoblastoma, Hepaticojejunostomy, Liver, Hepatic ducts, Stenting

## Abstract

The distorted anatomy of the porta hepatis can significantly complicate the process of portal dissection. In cases of large hepatoblastomas, the distortion of portal structures becomes pronounced, making it particularly difficult to manage inflow control effectively. This anatomical alteration not only complicates dissection but also heightens the risk of displacing nearby biliary structures. Such displacement increases the likelihood of inadvertent injuries to the bile ducts, which may require additional surgical interventions, such as hepaticojejunostomy, to restore biliary drainage. Furthermore, complex liver resections frequently result in an increased rate of morbidity, underscoring the importance of meticulous surgical technique. To minimize the risk of preventable duct injuries and the subsequent complications they can cause, it is crucial to adopt strategies that enhance identification and preserve the integrity of the biliary anatomy. This image vignette demonstrates a straightforward yet effective technique for duct cannulation. By employing this maneuver, surgeons can achieve better visualization and delineation of the ductal anatomy, ultimately reducing the likelihood of accidental injuries during surgery. This careful approach is key to improving surgical outcomes and minimizing postoperative complications.

Large hepatoblastomas often lead to extreme distortion of portal structures, making it challenging to control the inflow [1]. Additionally, the displacement of biliary anatomy increases the risk of unintentional injuries, which may necessitate further procedures such as hepaticojejunostomy [2]. Complex liver resections are associated with a corresponding increase in morbidity [3]. Therefore, it is essential to take measures to prevent duct injuries and the complications that arise from them. This image vignette demonstrates a simple maneuver for duct cannulation, which aids in better identifying and delineating the duct’s course, ultimately reducing the risk of accidental injury.

## Case Vignette

**Fig. 1:**
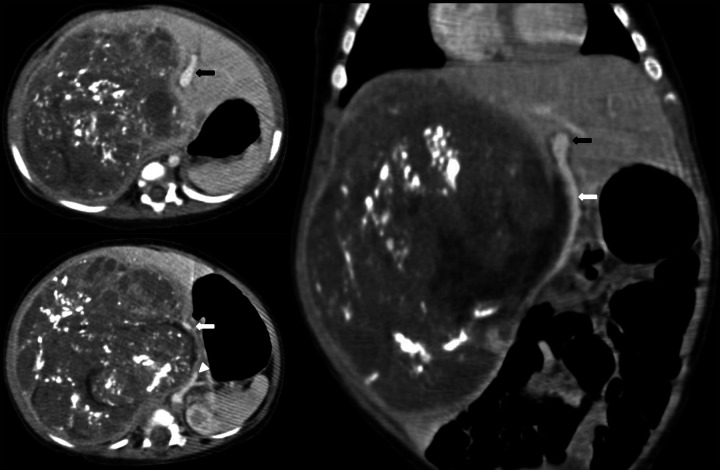
Post chemotherapy contrast-enhanced triphasic computed tomography, showing heterogeneously enhancing lesion with chunky calcification and necrotic non-enhancing spaces involving the entire right lobe with a large inferior exophytic component. The portal structures, aorta, and adjacent organs are grossly displaced by the mass. The main (white arrows), left branch of portal vein (black arrows), and common hepatic artery (arrow head) are stretched anteriorly along their medial edges while the right branch and inferior vena cava are not visible due to compression by the lesion

An 8-month-old child presented with an abdominal lump that had been present for the past month. A contrast-enhanced computed tomography (CECT) scan revealed massive hepatomegaly, with a 10.9 × 10.8 × 13.3 cm heterogeneously enhancing mass occupying the entire right lobe and the caudate lobe of the liver (Fig. 1). The mass showed extensive chunky calcifications, as well as necrotic and cystic hypodense areas. The inferior exophytic component displaced the portal structures anteriorly with splaying of the hepatic arteries and non-visualization of the right branch of portal veins. The aorta, celiac artery, and superior mesenteric artery were displaced along the medial border of the lesion, while the inferior vena cava (IVC) was compressed and not visible. Adjacent organs, including the gall bladder, kidney, and adrenal gland, were severely compressed and displaced. The alpha-fetoprotein (AFP) level was elevated to 860,000 ng/mL. Based on the radiological extent of the disease, the patient was categorized as having PRETEXT III disease with an annotation factor V + (venous) and stratified as having high-risk hepatoblastoma. The patient received seven cycles of SUPERPLADO regimen chemotherapy, which consisted of doxorubicin and alternating cycles of cisplatin and carboplatin over a period of 13 weeks. A follow-up CECT showed stable anatomical disease and a satisfactory biochemical response, with the AFP levels dropping to 171 ng/mL. A right extended hepatectomy was planned following a multidisciplinary meeting.

**Fig. 2:**
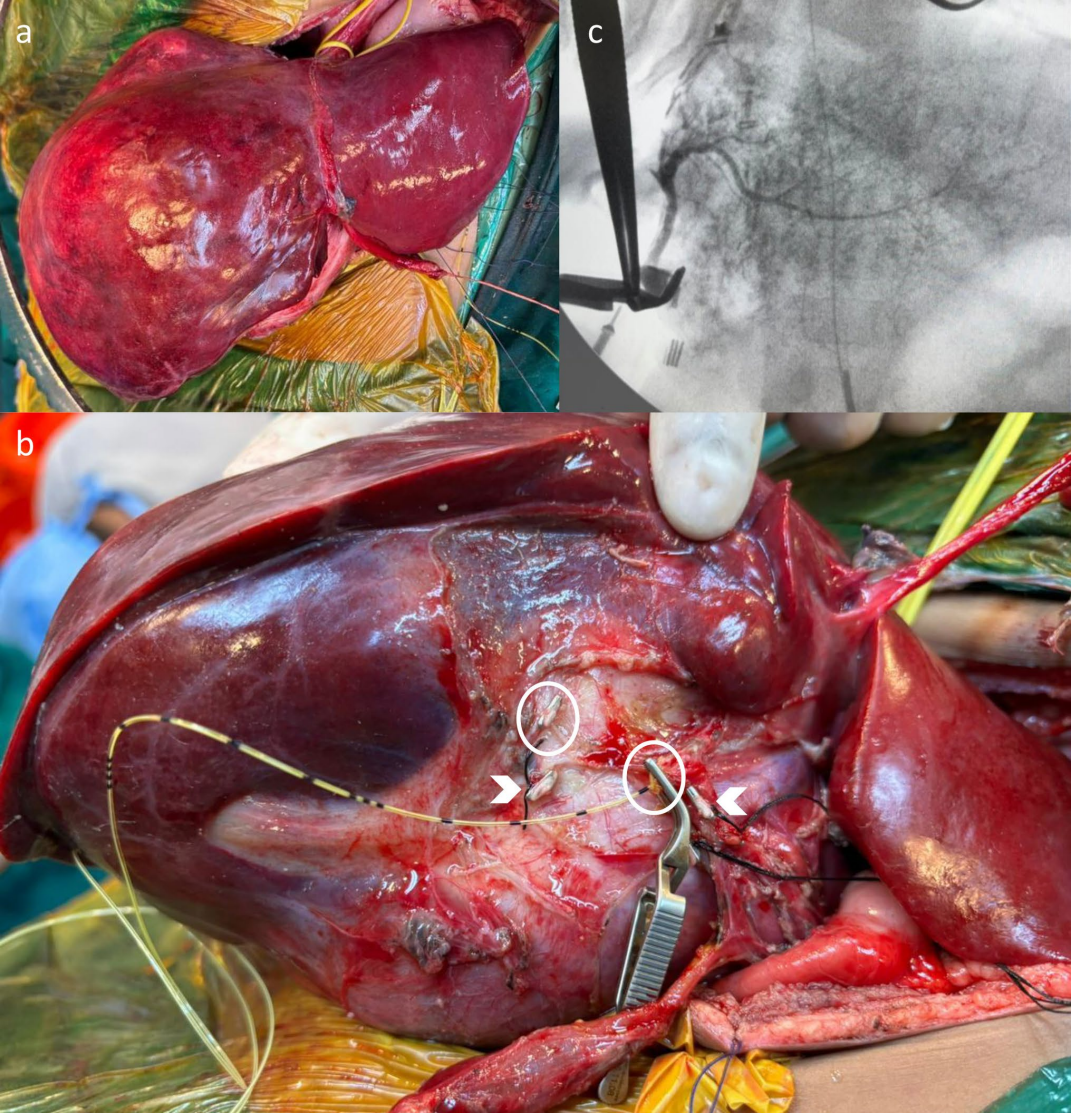
**a** The liver delivered outside the abdomen after complete mobilization; **b** the proximal and distal stumps of the right portal vein (arrow heads) and hepatic duct (circled) while the left portal vein is seen stretched and displaced by the underlying mass. The proximal stump of the right duct is cannulated using a 20-gauge epidural catheter as a tactile guide to dissect the left duct safely. **c** Intraoperative cholangiogram after right extended hepatectomy showing the duct draining the segments 2 and 3

A liver volumetry analysis conducted using Myrian software indicated a future liver remnant (FLR) of 72%. During the operation, the portal structures were not clearly defined, and the common hepatic duct appeared to be directly entering the liver mass. The right hepatic artery and the right branch of the portal vein were carefully dissected, looped, and divided flush with their entry into the mass. After the division of the right portal vein, the right hepatic duct was visualized. The hepatic duct confluence and the left hepatic duct were obscured and appeared flush, lacking clear demarcation on the under surface of segment 4b (Fig. 2). At this point, the right hepatic duct was cut close to where it entered the mass. The distal end of the right hepatic duct was cannulated using a 20-gauge epidural catheter. This stented duct served as a guide for the careful dissection of the left hepatic duct away from the liver lesion toward the left lateral sector. Tributaries from segment 4b were identified, ligated, and secured along this pathway. The main portal vein, appearing to continue as the left portal vein, was observed obscured in the umbilical fissure. It was gently dissected away from the under surface of the tumor. After achieving control over all inflow, a right extended hepatectomy was performed. Post-resection, an intraoperative cholangiogram revealed the left duct without any extravasation of contrast. The opening of the right duct was then ligated and secured. Hemostasis was confirmed, and the abdomen was closed with a drain placed in the surgical bed. Blood loss during the procedure was 300 mL. This report emphasizes the effectiveness of simple techniques, such as hepatic duct cannulation, to guide dissection and prevent inadvertent injuries, which could have necessitated a hepaticojejunostomy.

## Data Availability

The authors confirm that the data supporting the findings of this study are available within the article.
